# Bullying, minority stress and revenge impulse among autistic college students: group differences by sexual and gender minority status

**DOI:** 10.3389/fpubh.2026.1727508

**Published:** 2026-03-09

**Authors:** Wang Qian, Chunming Chen, Baojian Wei

**Affiliations:** 1 School of Teacher Education, Weifang University, Weifang, China; 2 Faculty of Humanities and Social Sciences, University of Nottingham, Ningbo, China; 3 School of Food and Health, Guangzhou City Polytechnic, Guangzhou, China; 4 School of Nursing, Shandong First Medical University & Shandong Academy of Medical Sciences, Jinan, China

**Keywords:** autistic university students, hostile attribution bias, internet use, mental health, minority stress, social bullying, social support, structural inequality

## Abstract

This study examines how bullying experiences are associated with retaliatory impulses among autistic university students, highlighting minority stress as a key mediating mechanism and sexual and gender minority (SGM) status as a moderating condition within an intersectional framework. Guided by Minority Stress Theory and Social Information Processing Theory, we surveyed 280 autistic undergraduates; 35% identified as SGM. Participants completed validated measures of bullying, minority stress, retaliatory impulse, and related psychosocial factors. Structural equation modeling and multi-group analyses were conducted to evaluate the proposed mediation and moderation patterns while adjusting for gender, grade level, social support, autistic traits, and internet use. The measurement model showed good reliability and convergent validity (Cronbach’s *α* = 0.84–0.89; AVE = 0.62–0.69). Bullying was positively associated with minority stress, which was in turn associated with retaliatory impulses, supporting partial mediation [indirect effect = 0.42, 95% CI (0.36, 0.50)]. Multi-group results indicated stronger path coefficients in the SGM group (bullying → stress *β* = 0.72; stress → retaliation *β* = 0.66) than among non-SGM (heterosexual and cisgender) peers, consistent with the possibility that compounded stigma heightens emotional reactivity and defensive processing. Minority stress remained the strongest correlate of retaliatory impulses after covariate adjustment, whereas social support showed a protective association. Taken together, the findings suggest that retaliatory impulses among autistic students are better understood in relation to sustained identity-based exclusion and structural stressors rather than as simple dispositional aggression. The results also imply that effective prevention may require institutional and relational strategies—alongside individual support—such as inclusive curricula, peer sensitization, and policies that strengthen belonging and psychological safety in higher education.

## 1 Introduction

In recent years, research on social adaptation and mental health among individuals with Autism Spectrum Disorder (ASD) in higher education has grown substantially. With the rise of the neurodiversity paradigm, scholars have increasingly shifted from a pathological deficit perspective toward one that recognizes autism as a form of neurological variation. This shift emphasizes that social environments should adapt to human diversity rather than require individuals to conform to normative expectations. Despite this theoretical shift, evidence consistently indicates that autistic university students experience disproportionately high levels of social exclusion, discrimination, and bullying within campus settings. Empirical studies across multiple countries have consistently shown that, in both offline and online settings, autistic students are more likely than their neurotypical peers to experience verbal aggression, social isolation, and cyber exclusion. The complex and loosely structured social systems of universities, characterized by implicit norms and flexible peer networks, further exacerbate this vulnerability. Autistic students are often misunderstood or marginalized due to atypical communication styles, differences in emotional expression, and limited access to implicit social rules. Accumulated experiences of bullying and rejection not only cause psychological harm but also undermine academic engagement, self-esteem, and a sense of belonging, leading to long-term mental health consequences.

Globally, empirical findings converge on the observation that students with ASD face significantly higher rates of bullying compared to their neurotypical counterparts, though manifestations vary across cultural and national contexts. In the United States, data from the Interactive Autism Network indicate that approximately 63% of autistic students report being bullied at least once during their education—three times the rate of their peers (1). A broader national analysis found that 46.3% of autistic adolescents had been bullied, 14.8% had engaged in bullying, and 8.9% had experienced both roles (2). Systematic reviews and meta-analyses across the U.S., U.K., Canada, and the Netherlands reveal victimization rates ranging from 7 to 75%, roughly two to three times higher than those of non-autistic students (3). In the United Kingdom, even in schools with anti-bullying frameworks, autistic students continue to face isolation linked to teachers’ limited sensitivity to neurodiverse behaviors (4). Australian and Canadian studies similarly report heightened bullying risks during the transition from high school to university, particularly where inclusive support systems are underdeveloped; up to 77% of autistic adolescents reported being bullied (5). Although Nordic countries lead in educational equality and neurodiversity advocacy, small-sample research in Finland and Norway has found that autistic students remain socially peripheral, with higher loneliness and alienation scores than neurotypical peers (6). In East Asia, Japanese qualitative studies show that autistic adults and students often face lifelong exclusion rooted in collectivist social norms that stigmatize behavioral deviation (7). Korean findings similarly suggest that conformity pressures and group norms limit peer acceptance for autistic students (8). In China, emerging evidence indicates that autistic college students experience social rejection both in dormitories and digital spaces, reflecting persistent public misconceptions about neurodiversity (9). Although research remains sparse in Latin America, South Asia, and Africa, studies on disability and sexual minority students suggest that bullying and stigma are more prevalent where institutional resources are scarce and cultural biases are strong (10). Taken together, evidence from North America, Europe, Oceania, Asia, and the Global South indicates that autistic students face consistently high social vulnerability worldwide. Cultural norms, social attitudes, and institutional inclusiveness determine whether such risks are overtly visible or subtly embedded, underscoring the structural nature of neurodivergent marginalization across societies.

From a theoretical standpoint, the roots of autistic students’ vulnerability can be traced to early cognitive and social theories. Traditional “individual deficit models” attributed social difficulties to impairments in theory of mind, emotion recognition, and pragmatic reasoning, implying that social exclusion stems from internal deficits. This framing inadvertently legitimized marginalization by overlooking structural and cultural factors. Subsequent studies revealed that autistic students demonstrate far greater social competence in inclusive and supportive environments, shifting scholarly discourse toward a “reciprocal exclusion cycle” framework, which conceptualizes social difficulties as co-constructed interactions rather than inherent impairments. The concept of “camouflaging,” wherein autistic individuals consciously suppress or mimic behaviors to conform socially, has further exposed the emotional cost of adaptation—often resulting in exhaustion, alienation, and identity confusion.

Within this evolving theoretical landscape, Minority Stress Theory (MST) has emerged as a powerful framework for understanding the chronic psychological strain experienced by marginalized groups. Originally proposed by Meyer (11) to explain stress processes among LGBTQ+ populations—often discussed under the broader umbrella of sexual and gender minorities (SGM)—MST distinguishes between distal stressors (e.g., discrimination, prejudice, and exclusion) and proximal stressors (e.g., internalized stigma, concealment, and expectations of rejection). Scholars such as Botha and Frost have extended this framework to “neurominorities,” arguing that autistic individuals experience structurally similar stress mechanisms. Yet, this extension also invites critical reflection: while it exposes systemic oppression, it risks reinforcing a victimizing narrative that diminishes autistic agency and resistance. Intersectional perspectives further complicate this picture, showing that when neurodivergent and gender or sexual minority identities overlap, psychological stress intensifies in nonlinear ways. Empirical studies confirm that perceived discrimination correlates negatively with mental health, with internalized stigma and identity concealment mediating this link (9). However, most such research has been conducted in Western contexts, leaving cultural collectivism and norm conformity underexplored. In East Asian and Latin American societies, where conformity and normative expectations are heavily institutionalized, autistic students experience heightened identity concealment stress and reduced willingness to seek support.

Prolonged exposure to discrimination and exclusion may lead to defensive cognitive processing patterns and hostile attribution biases, fostering retaliatory impulses. Research on bullying victims suggests that chronic rejection enhances rumination and moral disengagement, transforming anger into perceived justice restoration through retaliation. Such behaviors should not be pathologized but rather understood as expressions of agency within oppressive contexts. While social support, forgiveness, and adaptive coping can buffer these effects at the individual level, they are insufficient without structural inclusion. Hence, addressing autistic students’ bullying and psychological stress requires moving beyond deficit-based or intrapsychic explanations toward a structural analysis of power, inequality, and cultural normativity. In this sense, MST serves not merely as an explanatory tool but as a critical framework for unveiling systemic inequities, rethinking educational practices, and promoting neuroinclusive social transformation.

Overall, the bullying experiences and psychosocial vulnerabilities of autistic university students reveal not only cognitive or emotional differences but also the deep entanglement of social inequality and institutional exclusion. Across cultures and systems, evidence points to a persistent marginalization of autistic individuals within education. Integrating MST with Social Information Processing frameworks enables a dual-level understanding of both structural and cognitive mechanisms underlying autistic students’ mental health and behavioral responses. Moreover, adopting an intersectional lens highlights the compounded psychological burdens of multiple minority identities and advances the call for equitable, neuroinclusive university environments. Future cross-cultural research that empirically examines the pathway linking bullying experiences → minority stress → cognitive bias → retaliatory behavior would provide robust evidence for this integrative model and inform evidence-based interventions to foster inclusive higher education.

MST provides a central framework for understanding mental health among autistic and sexual minority populations. Meyer (11) argues that social prejudice and discrimination undermine mental health through the joint influence of distal stressors, such as structural discrimination, and proximal stressors, such as internalized stigma and identity concealment. This model has strong empirical support in explaining psychological distress among LGBTQ+ communities and offers a starting point for investigating mechanisms in other marginalized groups. Building on this work, Botha and Frost (12) extend the minority stress model to autism, showing that autistic individuals experience structural discrimination, social exclusion, and identity-related pressures. In contrast to traditional sexual minority stressors, autistic people also face internal processes linked to social communication challenges and self-appraisal biases. The extended model emphasizes the interaction between social structure and individual psychology and thus offers an integrated perspective on psychosocial vulnerability under multiple minority identities.

Within this framework, Salafia et al. (13) report that adolescents with both sexual minority and disability identities are more likely to experience bias-based bullying in schools, originating not only from peers but also from institutional practices that encode implicit discrimination. The compounded pressures associated with multiple minority statuses exert an amplifying effect, as forms of exclusion cumulate to produce heavier psychological burdens and stronger hostile affect. These findings supply key empirical groundwork for examining intersectional stress and behavioral responses among autistic SGM university students. Victimization through bullying functions as a major external trigger of minority stress. A longitudinal study by Xie et al. (14) shows that traditional, face-to-face victimization significantly predicts later cyberbullying perpetration, suggesting the development of a defensive aggression pattern under sustained social exclusion. This evolution reflects not only affective reactions but also shifts in cognitive processing. Victimization heightens sensitivity to hostile cues and threat perception, which activates a retaliatory processing pathway. From a Social Information Processing perspective, Mazzone et al. (15) further delineate the mechanisms linking bullying, moral disengagement, and retaliatory behavior. In contexts of social exclusion, individuals may develop attributional biases and moral-cognitive dissonance that normalize retaliation as a justified coping strategy. This theoretical account clarifies the cognitive basis for the mediating role of minority stress. For autistic and SGM University students, chronic discrimination and exclusion may intensify internalized pressures, activate hostile processing, and increase the likelihood of retaliatory impulses or aggressive intentions.

Drawing on these theoretical and empirical insights, the present study proposes an analytic framework in which bullying victimization leads to retaliatory impulse primarily through minority stress. The model integrates three levels of mechanism: social structure, psychological stress, and cognitive processing. Bullying victimization represents an external social stimulus. Minority stress captures the internalization of prejudice as stress responses. Retaliatory impulse reflects a behavioral tendency that emerges when cognitive processing becomes imbalanced. The framework explicates an intersectional stress chain and accounts for the emergence of defensive and hostile responses among autistic students under persistent social exclusion.

The study tests three hypotheses:

1. Bullying victimization will positively predict levels of minority stress.
2. Minority stress will mediate the association between bullying victimization and retaliatory impulse.
3. SGM status will moderate this mediating pathway, such that the indirect effect is stronger for SGM students than for non-SGM students.

## 2 Literature review

### 2.1 Social vulnerability and bullying risk among autistic university students

Individuals diagnosed with Autism Spectrum Disorder (ASD) often face profound difficulties in social interaction, communication, and emotional regulation, which make them particularly vulnerable to social exclusion and psychological strain in higher education contexts. This vulnerability extends beyond challenges in forming and maintaining peer relationships to encompass the cognitive and emotional burden of navigating complex social environments and interpreting ambiguous interpersonal cues. The university setting—characterized by diffuse social structures, implicit social norms, and diminished institutional guidance—magnifies these difficulties, leaving autistic students more susceptible to misunderstanding, stigmatization, and marginalization. Empirical studies, such as that of Kim et al. (16), have shown that autistic university students report higher rates of discrimination, harassment, and social exclusion than their neurotypical peers, reflecting the compounded influence of structural biases and informal peer exclusion. Although social support networks and inclusive educational policies can provide some protection, they are often insufficient to counteract the chronic stress generated by systemic barriers and social misunderstanding. Large-scale reviews indicate that autistic students are at a substantially greater risk of bullying across all educational stages, with the risk peaking in university environments where social hierarchies and expectations for self-regulation are more complex (17). Their difficulties in decoding social cues, non-normative communication styles, and sensory sensitivities often lead to misperceptions of them as aloof, odd, or uncooperative, which in turn intensify their marginalization and exposure to verbal aggression, exclusion, and cyberbullying. (37) found that autistic students are not only more likely to experience victimization but may also become inadvertently involved in conflicts due to misinterpreting others’ intentions or responding in atypical ways, underscoring how cognitive processing differences and communicative challenges interact to heighten social risk. Epidemiological findings by Junttila et al. (18) further demonstrated that social reciprocity deficits, emotional recognition difficulties, and rigid communication styles significantly predict bullying victimization, even beyond observable behavioral differences, suggesting that social misinterpretation and environmental bias jointly shape these experiences. The psychological consequences of bullying are particularly severe among autistic individuals. Lin et al. (19) identified strong associations between a history of peer victimization and increased depression, anxiety, and reduced self-efficacy, with social support and resilience serving only partial buffering roles. Limited social networks and emotional regulation difficulties constrain autistic students’ access to these protective factors, amplifying the long-term impact of exclusion and discrimination. Persistent marginalization fosters rumination, social withdrawal, and avoidance behaviors, which further diminish social engagement and reinforce psychological distress. As digital communication becomes integral to academic and social life, new vulnerabilities emerge; DiRienzo et al. (20) reported that autistic university students frequently encounter targeted hostility online, where anonymity and rapid dissemination exacerbate harm and make intervention more difficult. Their communication patterns—often literal, direct, or emotionally atypical—are easily misinterpreted as rudeness or detachment, perpetuating cycles of misunderstanding and exclusion. Similar dynamics are observed among other marginalized groups. Gower et al. (38) noted that LGBTQ youth experience disproportionate bullying linked to gender nonconformity and heteronormative expectations, reflecting shared mechanisms of prejudice and structural discrimination. For autistic students who also identify as gender or sexual minorities, such intersectional vulnerabilities intensify stigmatization, heighten isolation, and compound psychological strain. The social fragility of autistic university students thus arises from the intersection of neurocognitive differences and institutional structures that fail to accommodate them adequately; ongoing discrimination, limited inclusion, and inadequate systemic support transform everyday interactions into sources of sustained stress, impeding their academic participation, social belonging, and overall mental well-being while underscoring the urgent need for neuroinclusive practices within higher education.

### 2.2 MST and the intersectional identity framework

Minority Stress Theory (MST), first proposed by Meyer, serves to elucidate the causal mechanisms linking structural discrimination to mental health disparities. It emphasizes that individuals occupying minority positions endure not only general life stressors but also additional distal stressors such as social prejudice, discriminatory incidents, and systemic exclusion, alongside proximal stressors including internalized stigma, identity concealment, and expectations of rejection (21). These cumulative pressures exert continuous influence on cognitive and emotional processes, resulting in sustained psychological strain and social–cognitive distortions. In recent years, MST has been widely applied within the study of intersectional identities, shedding light on the intricate interplay among multiple marginalized statuses. Sarno et al. (22) demonstrated that when individuals simultaneously possess both sexual and racial minority identities, their psychological distress is not a simple additive outcome; rather, it emerges from the intersection of identity conflict, social expectations, and belonging uncertainty, forming a distinct pattern of structural vulnerability. This finding has shifted scholarly discourse from single-dimensional stress interpretations toward a more systemic and multilayered intersectional minority stress framework. From a micropsychological perspective, (39) revealed that subtle discrimination and microaggressions can indirectly heighten depressive symptoms by undermining self-esteem and perceived control, underscoring the buffering role of positive self-concept and social support networks. Building upon this foundation, Rivas-Koehl et al. (23) introduced the Temporal Intersectional Minority Stress Model (TIMSM), which posits that minority stress evolves dynamically over time, as individuals’ perceptions of identity and social acceptance adjust across developmental stages. This model is particularly pertinent to university students—such as autistic and SGM populations—who are engaged in processes of identity formation and negotiation. Empirical evidence from Adeyeba et al. (24) further demonstrated that identity centrality and resilience moderate the relationship between stress and mental health, indicating that individuals who achieve a coherent integration of multiple identities are better equipped to regulate emotions and recover from adversity. These findings collectively suggest that intersectional minority individuals engage in active meaning-making rather than passive adaptation.

Within this theoretical context, autistic and SGM populations exhibit shared structural patterns of psychological vulnerability. Chaudoir et al. (25) noted that persistent exposure to prejudice and stigmatization erodes psychological functioning, manifesting as heightened anxiety, depression, self-denigration, and social withdrawal. For autistic university students, the coexistence of neurodivergent and sexual minority identities compounds sources of social exclusion and internal conflict, giving rise to a complex form of intersectional minority stress. Ferbežar et al. (26) found that LGBTQ+ students frequently experience identity concealment and internalized stigma in educational settings, where heteronormative and binary gender assumptions intensify identity management burdens. For autistic students, communicative challenges and emotional hypersensitivity exacerbate these effects, restricting access to peer support and amplifying psychological strain. Extending MST to neurodivergent populations, Botha and Frost (12) introduced the notion of “neurominorities,” emphasizing that autistic individuals face intertwined distal and proximal stressors—external exclusion rooted in social bias and internal conflicts arising from self-stigma—forming cumulative psychological loads. Evidence from Li et al. (9) based on Chinese university samples showed that perceived discrimination negatively predicts mental health, with internalized stigma mediating and social support moderating this relationship. However, for autistic students, limited social networks and communication barriers weaken the protective effects of social support, magnifying the adverse impact of minority stress on emotional and behavioral outcomes. Stańczykiewicz and Senczyszyn (27) further highlighted that gender norms and implicit biases embedded within school culture perpetuate discriminatory bullying structures and diminish students’ perceived safety and belonging, prompting avoidance, masking, and social camouflage as adaptive responses. This sustained psychological regulation exhausts emotional resources, fosters social withdrawal, and undermines academic engagement and resilience. Collectively, the expansion of MST within an intersectional framework illuminates the multidimensional interplay among social structures, individual cognition, and temporal development. Autistic and SGM individuals, persistently marginalized within social hierarchies, experience psychological stress not as a reflection of personal frailty but as a consequence of cultural prejudice, institutional discrimination, and systemic exclusion. Integrating MST with an intersectional perspective thus offers a more comprehensive understanding of the psychosocial challenges faced by autistic university students with multiple nonnormative identities, providing a robust theoretical foundation for fostering inclusivity and psychological safety in higher education contexts.

### 2.3 Psychological mechanisms of retaliatory impulses: a social information processing perspective

The Social Information Processing (SIP) theory provides a comprehensive cognitive framework for explaining how aggressive and retaliatory behaviors emerge and persist within social contexts. Originally proposed by Dodge and Crick (28), the theory conceptualizes social behavior as a multistage process involving the encoding of cues, interpretation of intent, generation of potential responses, and selection of behavioral action. Within this framework, aggression is not understood as a mere impulsive reaction but as the outcome of biased or maladaptive cognitive processing. When individuals misinterpret ambiguous social information—particularly through mechanisms such as hostile attribution bias or moral disengagement—they are more likely to perceive benign cues as threatening and to justify aggressive responses. Dodge and Rabiner (29) extended this model to emphasize that repeated victimization and chronic exclusion reinforce these cognitive biases, leading to a process of “cognitive consolidation” in which negative experiences heighten vigilance and defensive responding. The Dual-Mode SIP Model proposed by Verhoef et al. (30) further distinguishes between a rapid emotional route, which operates automatically under the influence of affective arousal, and a reflective cognitive route, which involves deliberate moral reasoning and consequence evaluation. Individuals with higher impulsivity tend to rely more on the emotional route, whereas those with stronger self-regulatory abilities can inhibit revenge-oriented impulses. For autistic university students, SIP distortions are particularly salient due to inherent challenges in decoding social cues, understanding emotions, and interpreting nuanced interpersonal contexts. These difficulties increase the likelihood that ambiguous signals will be perceived as hostile, thereby activating defensive or retaliatory behavioral tendencies (31). In this sense, SIP mechanisms explain not only the elevated risk of victimization among autistic individuals but also the potential emergence of retaliatory motives as a psychological response to perceived social threat.

Recent extensions of SIP theory further integrate developmental, emotional, and sociocultural dimensions to explain how prolonged social stress fosters aggression and revenge motivation. Vagos et al. (32) demonstrated that early maladaptive schemas and experiences of social rejection distort cognitive representations of interpersonal trust, predisposing individuals to interpret ambiguous interactions as threatening and to rely on retaliatory strategies to restore a sense of control and justice. For autistic university students, who often experience social exclusion and limited emotional support, this imbalance in cognitive-emotional processing can escalate into overt conflict or online aggression. Moral cognition is central to this trajectory. As Dodge and Rabiner (29) observed, when individuals employ moral disengagement—justifying aggressive acts as legitimate or deserved—they weaken internalized moral standards and normalize retaliation. This process becomes especially pronounced under minority stress conditions, where chronic exposure to stigma and injustice erodes emotional regulation and moral constraint, transforming aggression into a form of psychological compensation. Empirical studies confirm these pathways: Saricam and Cetinkaya (33) found that revenge motives mediate the link between bullying victimization and subsequent perpetration, while (40) revealed that hostile attribution bias and rumination explain how peer victimization predicts revenge intentions. Longitudinal evidence by Xie et al. (14) indicates that traditional bullying can evolve into online aggression, particularly when anonymity and social disinhibition lower the thresholds for retaliatory behavior. Ding et al. (34) emphasized the role of trait anger and revenge motivation in this process, noting that digital environments amplify latent hostility by diminishing moral constraints. Conversely, Sechi et al. (35) highlighted forgiveness as a protective factor that fosters emotional reappraisal and reduces the drive for retaliation. Collectively, these findings position SIP theory as a critical bridge between individual cognition and social experience, explaining how persistent injustice and exclusion are internalized through distorted social perception and externalized through defensive aggression. For autistic and SGM university students, whose lived experiences intertwine chronic victimization with structural discrimination, the integration of SIP theory and Minority Stress frameworks provides a robust explanatory lens for understanding how systemic inequity translates into cognitive imbalance, emotional strain, and retaliatory behavioral outcomes.

### 2.4 An integrated model of bullying, minority stress, and retaliatory impulses

Bullying victimization is a persistent source of social stress in the university years, and it is especially destabilizing—emotionally and cognitively—for autistic and sexual minority students. According to a systematic review by Wang and Susumu (17), differences in nonverbal communication, social cue interpretation, and interpersonal negotiation place autistic undergraduates at a disadvantage in peer relations. Experiences of exclusion or misunderstanding often consolidate into a chronic sense of social threat, increasing the tendency to adopt defensive or retaliatory strategies to preserve psychological safety in subsequent situations. Under intersectional identities, minority stress arises not only from external discrimination but also from tensions within the identity system itself. Adeyeba et al. (24) note that the overlap of gender, SGM status, and neurodivergent identities exposes individuals to multiple, simultaneous evaluative standards. To mitigate risks of exposure and stigma, they repeatedly adjust self-presentation across contexts. This prolonged defensive stance erodes emotion regulation and identity coherence, making social stimuli more readily construed as threatening and thereby strengthening aggressive inclinations.

The link between bullying and retaliation depends heavily on patterns of cognitive and affective processing. In a systematic review, (47) show that post-victimization emotional responses are shaped by cognitive regulation strategies. Rumination, self-blame, and catastrophizing foster a sustained expectation of threat, making aggression or revenge more likely as an outward sign of dysregulated control. By contrast, positive reappraisal and social support dampen affective volatility and reduce the probability of aggressive intent. (41) further specify this chain by demonstrating that maladaptive cognitive–emotional regulation mediates the association between bullying and traumatic symptoms, and that hope attenuates this pathway. Individuals high in hope tend to retain goal focus and a sense of agency when facing adverse events, which reduces retaliatory thinking. Psychological resources thus moderate the stress process by helping victims maintain cognitive stability and behavioral restraint in threatening contexts. Affective support networks are equally critical moderators. Zhang et al. (36) find that emotional support from teachers and families significantly lowers the risk of self-harm and hostile reactions after victimization by enhancing emotional safety and social trust. In supportive interpersonal milieus, individuals interpret social cues through less hostile lenses, weakening the cognitive foundations of revenge. An inclusive institutional climate therefore shapes not only the pace of psychological recovery but also the direction and quality of social information processing. Within this integrated framework, bullying activates external stressors, minority stress drives ongoing emotional depletion, and cognitive-processing biases inflate threat perception, while social support and psychological resources counteract these cumulative effects at multiple stages. Autistic and SGM undergraduates exhibit heightened sensitivity and stronger responses within this system. Constraints in social cognition and emotion regulation make them more susceptible to a cycle that progresses from victimization to defense and, ultimately, to retaliation.

## 3 Methodology

### 3.1 Research design and theoretical framework

**Figure 1:**
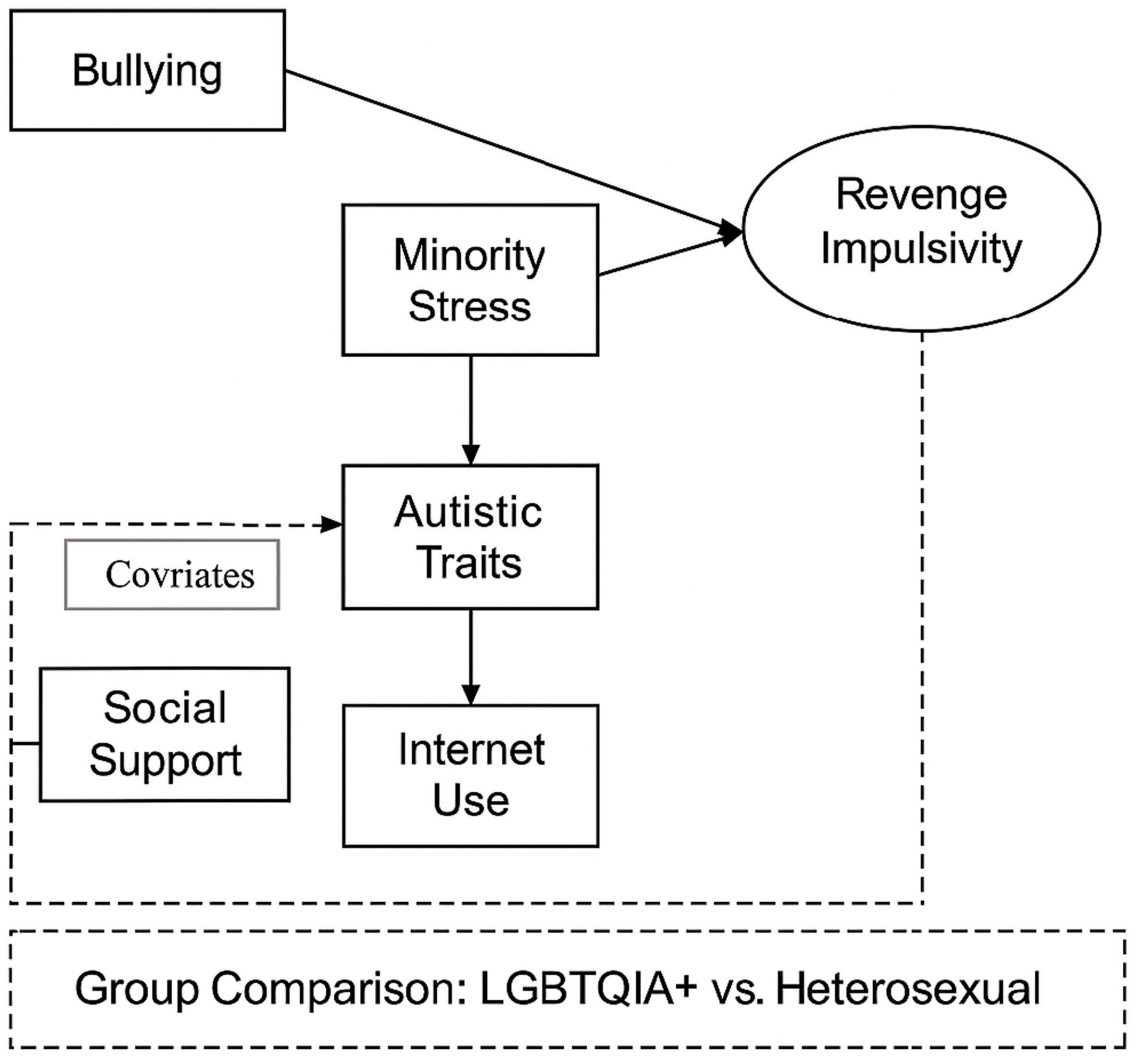
Theoretical framework and hypothesized model of bullying, minority stress, and revenge impulsivity.

This study employs a cross-sectional quantitative design and uses Structural Equation Modeling (SEM) as the primary analytic approach to examine the mechanisms linking psychological stress and retaliatory impulses among autistic university students following traditional and cyberbullying. The model also compares pathway differences between SGM and heterosexual students in order to clarify how minority stress operates under intersectional identities and what this implies for social adaptation. We hypothesize that bullying increases minority stress, which heightens emotional vulnerability and psychological disequilibrium, thereby fostering the emergence and maintenance of retaliatory impulses. Social support, autistic traits, and intensity of internet use are incorporated to test the moderating and buffering roles of environmental and individual factors. By modeling these variables jointly, the study aims to build an integrative framework that captures stress responses and behavioral outcomes for neurodivergent students in contexts of social exclusion (see Figure 1).

The model rests on the Minority Stress Model (11), the General Aggression Model (42), and Intersectionality Theory (43). The Minority Stress Model posits that chronic exclusion, stigma, and discrimination produce internalized stress responses that erode emotion regulation and psychological resources. The General Aggression Model explains how external provocation and internal affective imbalance jointly shape impulsive behavior. Intersectionality Theory highlights the compounded vulnerabilities created by the overlap of SGM status and neurodivergent status, which introduces additional psychological challenges and structural barriers in coping with social exclusion. Taken together, these perspectives lead us to conceptualize retaliatory impulses as defensive responses arising from structural inequality and accumulated stress rather than as mere individual deviations.

The model further introduces a digital media environment component to examine how online engagement influences emotion regulation and social feedback, assessing whether virtual interaction amplifies or mitigates offline stress. This integrated framework refines theoretical accounts of mental health among neurodiverse and gender–sexual diverse populations and offers empirical leverage for understanding how social inequality is reproduced through psychological mechanisms.

### 3.2 Sample and data collection

Data were collected via Wenjuanxing, a major Chinese online survey platform, using a stratified convenience sampling strategy with gender and SGM status as the primary stratification variables, to ensure diversity and heterogeneity in social identity and psychological characteristics. SGM status and gender identity were collected as separate self-reported demographic indicators and were used solely for sampling stratification and classification purposes. The target population comprised enrolled university students who self-identified as autistic or reported autistic traits. Eligibility criteria were:

(1) Current enrollment in a university program;
(2) At least three experiences of, or exposures to, on-campus bullying or cyberbullying in the past 3 months; and
(3) Voluntary participation with complete survey responses. To ensure data quality, cases with abnormal completion times, logical inconsistencies, or duplicate IP addresses were excluded.

After screening, 280 valid responses were retained (validity rate = 81.2%). The sample included 137 men (48.9%) and 143 women (51.1%); based on reported SGM status and/or gender identity, 98 participants (35.0%) were classified as sexual and gender minorities (SGM), whereas the remaining 182 participants (65.0%) were categorized as non-SGM (heterosexual and cisgender). Ages clustered between 18 and 25 years and spanned all undergraduate cohorts. All participants provided informed consent prior to responding. The anonymous questionnaire required approximately 12 min to complete. It should be noted that SGM status was not treated as an explanatory variable in the theoretical model but served as one component of the operational definition of SGM status. The strict inclusion and exclusion criteria, together with the stratified sampling approach, support a diverse sample suitable for subsequent structural equation modeling analyses.

### 3.3 Indicator system

Table 1 shows theoretical dimensions, operational definitions, and measurement instruments for the main study variables.

**Table 1:** Theoretical dimensions, operational definitions, and measurement instruments for the main study variables.

Primary construct (theoretical dimension)	Secondary construct (measurement dimension)	Observable variable/operational definition
Bullying experiences	Cyberbullying	Experiences of insult, exclusion, threats, doxxing, or hate speech on online platforms
	School bullying	Verbal, social, or physical exclusion and attacks occurring at school
Minority stress	Internalized stigma	Negative self-evaluation, self-blame, and shame related to autism or SGM identity
	Perceived discrimination	Degree to which one perceives discrimination, exclusion, or unfair treatment due to identity
	Identity disclosure stress	Anxiety, tension, and psychological burden associated with hiding or disclosing one’s identity
Retaliatory impulse	Affective revenge tendency	Anger and urges to retaliate when facing bullying incidents
	Intention to retaliate	Willingness to plan or enact retaliatory behaviors in offline or online contexts
SGM status identity	Orientation category	Self-identification as SGM or heterosexual
	Gender identity	Self-identified gender, for example man, woman, transgender, non-binary
Control variables	Social support	Perceived emotional and instrumental support from family, friends, and significant others
	Autistic trait severity	Level of expression of autistic characteristics
	Intensity of internet use	Daily online time, number and frequency of social media platforms used
	Demographics	Age, gender, year in school, family background, and related information

## 4 Results

### 4.1 Measurement model

The measurement model demonstrated strong reliability and convergent validity (*α* = 0.84–0.89; CR = 0.86–0.91; AVE = 0.62–0.69), suggesting a coherent and stable structure across all latent constructs. Yet, beyond its methodological robustness, these findings reflect a deeper psychological and social reality. The high internal consistency does not merely confirm psychometric soundness but reveals the shared psychological patterns shaped by long-term exposure to discrimination and identity-related stress. Such stability indicates that participants may have developed collective cognitive and emotional responses to persistent social marginalization. The reliability of constructs like perceived discrimination, internalized stigma, and retaliatory intention therefore represents not only consistent measurement but also the psychological imprint of structural inequality. For autistic and SGM students, continuous exposure to exclusion and prejudice appears to consolidate defensive attributional patterns—interpreting ambiguous social cues as threats and transforming emotional exhaustion into controlled forms of resistance. From this perspective, the strong correlations between minority stress and retaliatory impulse should not be read as individual pathology but as an adaptive response to chronic injustice. This interpretation underscores the theoretical tension between psychological resilience and social vulnerability: the same mechanisms that sustain emotional survival also perpetuate cognitive defensiveness. Consequently, the significance of these results extends beyond measurement validation. They highlight how enduring inequities become embedded in cognitive processing and emotional regulation, reminding scholars and institutions that meaningful intervention must go beyond individual coping skills to address the systemic roots of stress and retaliation within educational and cultural contexts (see Table 2).

**Table 2:** Reliability and validity testing results for the measurement model.

Latent variable	Items	Cronbach’s *α*	CR	AVE
Traditional bullying (CB)	5	0.87	0.89	0.64
Cyber bullying (SB)	4	0.85	0.88	0.62
Internalized stigma (IS)	4	0.88	0.9	0.67
Perceived discrimination (PD)	4	0.89	0.91	0.69
Outness stress (OS)	3	0.84	0.86	0.63
Revenge tendency (RT)	4	0.86	0.88	0.65
Revenge intention (RI)	4	0.85	0.87	0.64

### 4.2 Descriptive statistics

Our sample included 280 autistic undergraduates with balanced distributions by gender, year in school, and SGM status, but the pattern itself invites interpretation rather than simple description. The near parity between male and female participants and the presence of a small nonbinary subgroup suggest shifting pathways into diagnosis and disclosure in higher education, where service access, clinical gatekeeping, and changing cultural norms can modify who is visible as autistic on campus. The even spread across years implies that attrition is not disproportionately removing certain students from the pipeline, yet it may also reflect survivorship within institutions that still place a premium on conformity to implicit social rules. The proportion of SGM students is substantial, which is consistent with evidence that gender and sexual diversity are more common in autistic populations, but it may also be amplified by self selection, community networks, and a greater willingness among minoritized students to participate in research that promises recognition of stigma and stress. This configuration has theoretical and methodological consequences. It places intersectionality at the center rather than at the margin, implying that minority stress processes are not add-ons but constitutive features of the university experience for many autistic students. It also cautions that measurement invariance and structural paths in the bullying to minority stress to retaliation model must be interrogated for subgroup specific dynamics, since the meaning of discrimination, outness, and self protection can differ across identity constellations. Most importantly, the sample structure signals that what institutions label as diversity is often carried by students who shoulder the psychological costs of navigating doubly or triply non normative identities. The data therefore do not simply enable robust statistics; they reveal how universities curate belonging, how support systems may reach some groups while missing others, and how patterned exposure to prejudice becomes embedded in everyday appraisal and coping (see Table 3).

**Table 3:** Descriptive characteristics of the sample.

Variable	Category	*n*	%
Gender	Male	132	47.1
	Female	142	50.7
	Non-binary	6	2.1
Grade	Freshman	69	24.6
	Sophomore	74	26.4
	Junior	71	25.4
	Senior	66	23.6
SGM status	SGM	98	35
	Non-SGM	182	65

### 4.3 Group comparisons

**Figure 2:**
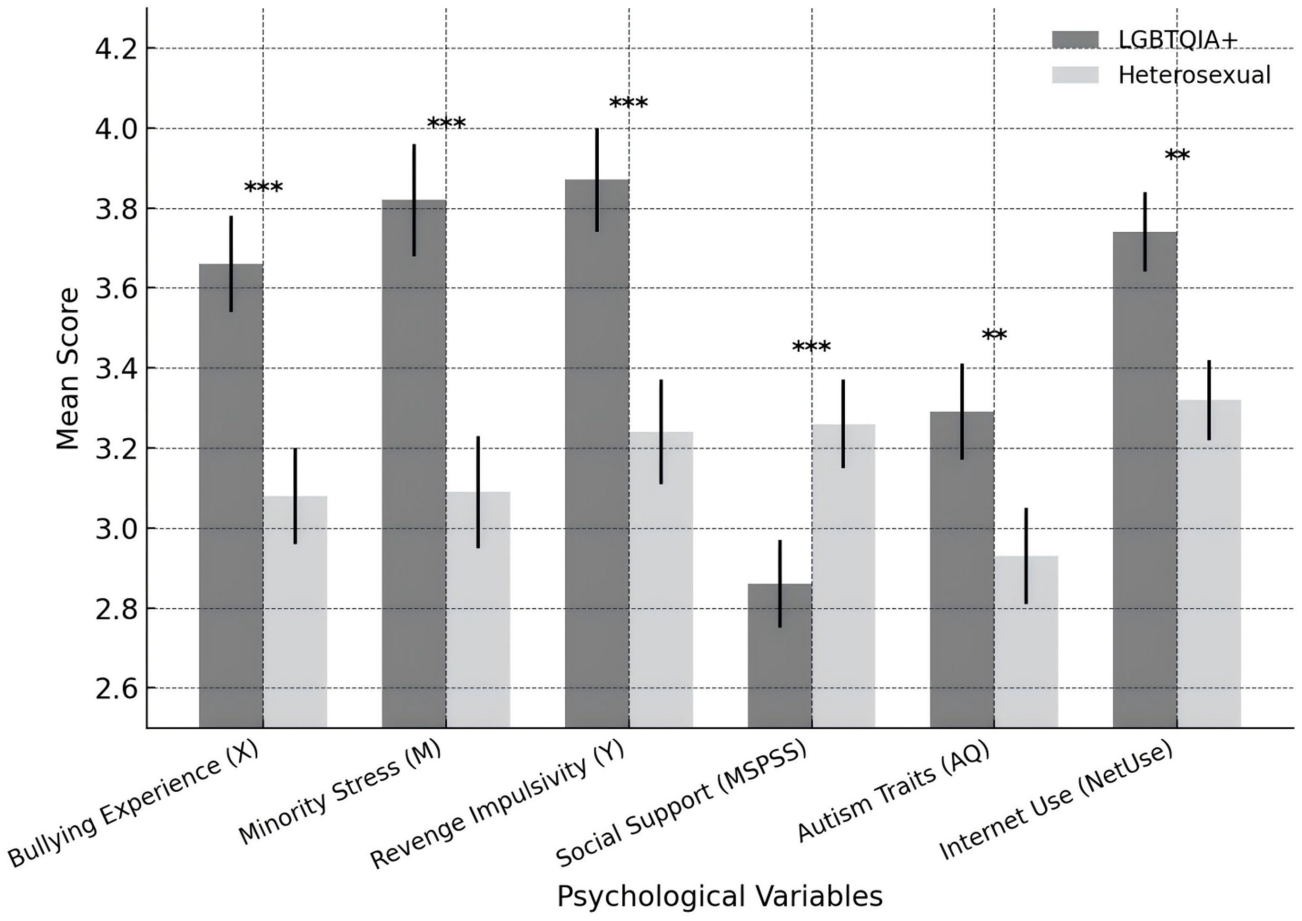
Group differences in key psychological variables by SGM status.

This study examined and interpreted the group differences between SGM and heterosexual autistic university students across key psychological variables, as summarized in Table 4, Figure 2. Overall, significant differences emerged in bullying experiences, minority stress, retaliatory impulse, social support, autistic traits, and online engagement (*p* < 0.001), yet the theoretical implications underlying these differences are more revealing than the numerical contrast itself. SGM students reported higher levels of bullying (3.66 vs. 3.08, *t* = 6.22, *p* < 0.001) and minority stress (3.82 vs. 3.09, *t* = 7.58, *p* < 0.001), suggesting that the intersection of SGM status and neurodivergent identity transforms social exclusion from an occasional event into a structural exposure. These findings do not merely indicate heightened sensitivity but rather reflect the cumulative psychological cost of managing marginalized identities and navigating unequal power relations within academic and digital environments. Their stronger retaliatory impulses (3.87 vs. 3.24, *t* = 6.93, *p* < 0.001) should not be seen as increased aggression but as a defensive cognitive pathway developed in response to chronic deprivation of fairness and agency—a self-protective adaptation to persistent injustice. At the same time, the significantly lower level of social support among SGM students (2.86 vs. 3.26, *t* = −4.02, *p* < 0.001) points to an inequitable distribution of emotional and relational resources, which may accelerate the psychological transmission from stress to impulsivity by limiting opportunities for emotional recovery. Their higher scores in autistic traits (3.29 vs. 2.93, *t* = 3.23, *p* = 0.001) may amplify sensitivity to ambiguous social cues and foster defensive interpretations, while increased online activity (3.74 vs. 3.32, *t* = 3.48, *p* = 0.001) reveals a dual pattern of seeking belonging and facing digital vulnerability. Thus, the patterns depicted in Figure 2 are not isolated statistical outcomes but reflections of how intersectional identities are shaped by institutional environments. These findings highlight that inclusion policies limited to symbolic destigmatization fail to address deeper inequities embedded in campus interactions, discourse, and grievance systems. When uncorrected, minority stress becomes a stable cognitive-emotional schema, and retaliatory tendencies are reproduced as adaptive responses to persistent imbalance. Therefore, institutional interventions must focus on rebuilding equitable relationships and safe contexts—through inclusive pedagogical practices, accessible psychological services, supportive peer communities, and effective digital governance—to reduce the structural roots of victimization and reshape the psychological trajectory from stress toward resilience and restored agency.

**Table 4:** Mean differences between SGM and Non-SGM (heterosexual and cisgender) students.

Variable	SGM (*n* = 98)	Non-SGM (*n* = 182)	*t*	*p*	Sig
Bullying experience (X)	3.66 (0.74)	3.08 (0.80)	6.22	<0.001	***
Minority stress (M)	3.82 (0.71)	3.09 (0.83)	7.58	<0.001	***
Revenge impulsivity (Y)	3.87 (0.79)	3.24 (0.77)	6.93	<0.001	***
Social support (MSPSS)	2.86 (0.74)	3.26 (0.77)	−4.02	<0.001	***
Autism traits (AQ)	3.29 (0.71)	2.93 (0.73)	3.23	0.001	**
Internet use intensity (NetUse)	3.74 (0.82)	3.32 (0.80)	3.48	0.001	**

*p < 0.10, **p < 0.05, ***p < 0.01.

### 4.4 Mediation analysis

**Figure 3:**
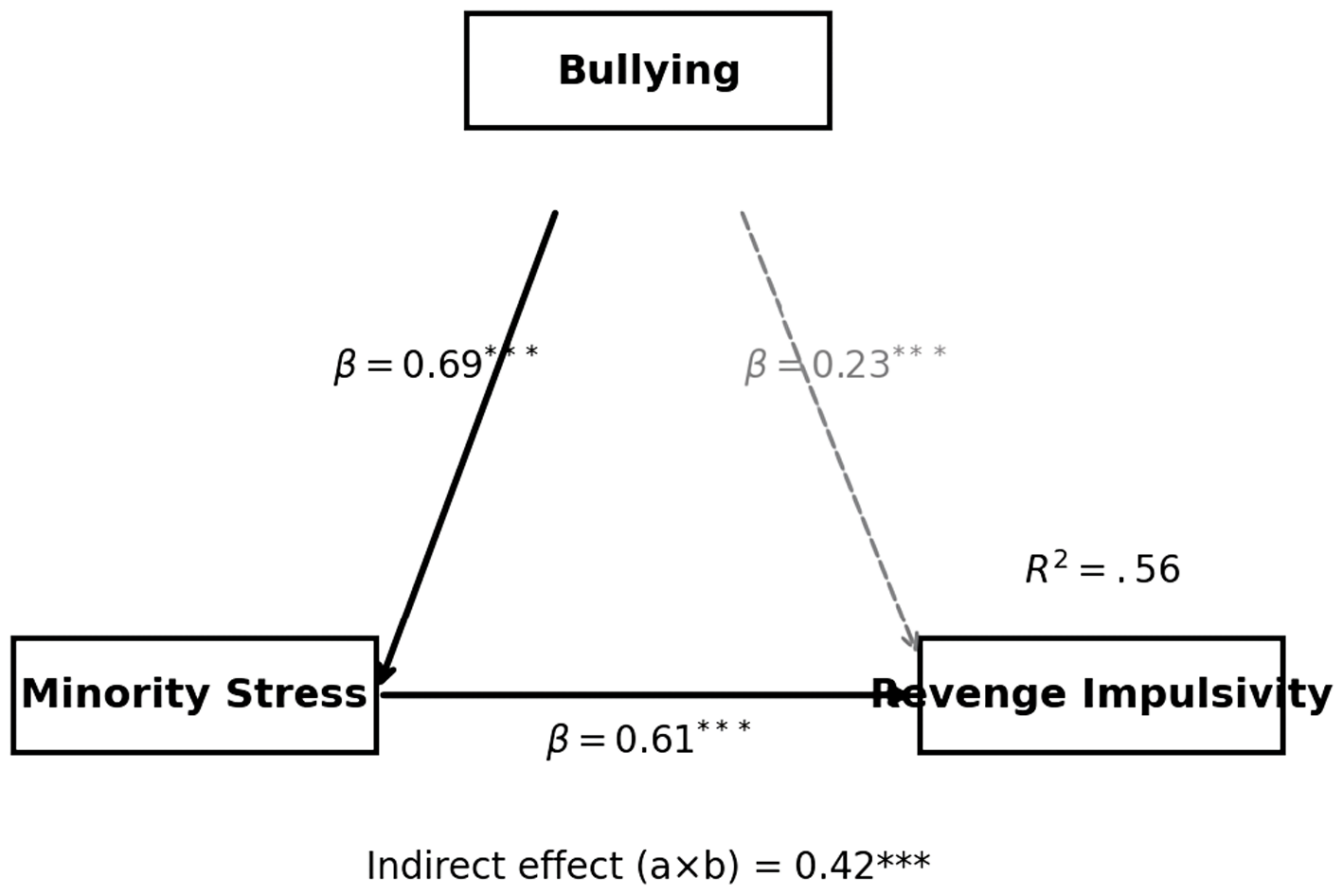
Structural equation model illustrating the mediating effect of minority stress.

This study further examined the mediating role of minority stress in the relationship between bullying experiences and retaliatory impulses, with the results presented in Table 5, Figure 3. The model showed good overall fit (*R*^2^ = 0.56), and all path coefficients reached significant levels (*p* < 0.001), indicating a stable psychological structure. Specifically, bullying experiences significantly predicted minority stress (*β* = 0.69, *t* = 12.48), suggesting that prolonged social exclusion accumulates into internalized stigma, perceived discrimination, and identity-related anxiety—forms of psychological strain that reflect the enduring imprint of structural inequality rather than transient emotional reactions. Minority stress, in turn, significantly predicted retaliatory impulses (*β* = 0.61, *t* = 11.22), showing that under conditions of high social pressure and marginalization, individuals are more likely to adopt hostile or defensive reactions as a means of regaining psychological balance and agency. Although the indirect effect was substantial (*a* × *b* = 0.42, accounting for roughly two-thirds of the total effect), the direct effect of bullying on retaliatory impulses remained significant (*β* = 0.23, *t* = 4.82), implying that retaliation arises from both immediate emotional responses and long-term internalization of social oppression. In essence, retaliatory tendencies are not merely impulsive acts but represent a defensive adaptation to persistent injustice and identity denial. This mechanism demonstrates that minority stress is not only a psychological construct but also a product of social reproduction. For SGM autistic undergraduates, prolonged stigmatization and exclusion result in both social isolation and emotional deprivation, with retaliatory impulses reflecting the tension between passive resistance and the reconstruction of self-agency. It is concerning that current university anti-bullying measures primarily target behavioral control while neglecting the continuous psychological stress experienced by intersectional minority groups, leading to the accumulation of emotional strain that may manifest through aggression-related schemas. The findings therefore suggest that effective intervention should extend beyond behavioral regulation to address psychosocial and institutional factors—reducing bias, minimizing identity exposure anxiety, and strengthening peer support and inclusion—to weaken the mediating effect of minority stress on retaliatory impulses and foster an academic environment characterized by structural equity and psychological safety.

**Table 5:** Mediation model results (direct, indirect, and total effects with 95% CI).

Path	*β*	*t*	*p*	95% CI	Sig
Bullying → minority stress (*a*)	0.69	12.48	<0.001	[0.58, 0.77]	***
Minority stress → revenge impulsivity (*b*)	0.61	11.22	<0.001	[0.51, 0.70]	***
Bullying → revenge impulsivity (direct *c*′)	0.23	4.82	<0.001	[0.14, 0.32]	***
Indirect effect (*a* × *b*)	0.42	—	<0.001	[0.36, 0.50]	***
Total effect (*c*)	0.65	—	<0.001	[0.55, 0.74]	***

*p < 0.10, **p < 0.05, ***p < 0.01.

### 4.5 Moderation and multi-group analysis

#### 4.5.1 Moderation by SGM status

**Figure 4:**
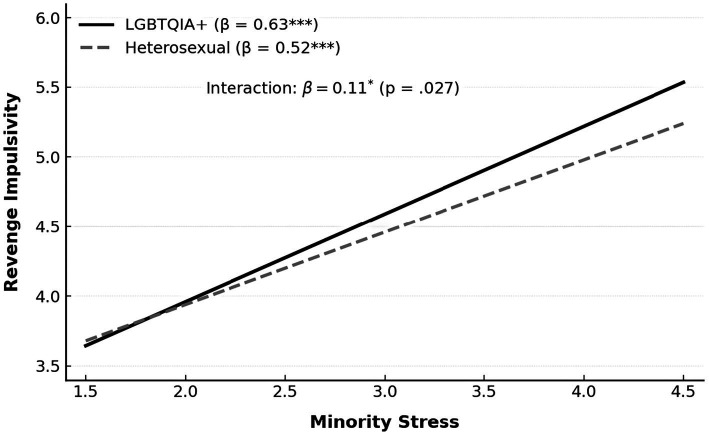
Moderating effect of SGM status on the link between minority stress and revenge impulsivity.

This study explored the mediating effect of minority stress on the relationship between bullying experiences and retaliatory impulses, as summarized in Table 5, Figure 3. The model fit was satisfactory (*R*^2^ = 0.56), with all paths reaching statistical significance (*p* < 0.001), suggesting a coherent and stable psychological structure. Bullying experiences strongly predicted minority stress (*β* = 0.69, *t* = 12.48), indicating that continuous social exclusion is internalized as stigma, perceived discrimination, and identity-related anxiety—psychological responses that reveal the persistent imprint of structural inequality rather than momentary emotional distress. Minority stress also significantly predicted retaliatory impulses (*β* = 0.61, *t* = 11.22), suggesting that when individuals experience sustained marginalization, they are more likely to develop defensive or oppositional tendencies as a way of restoring psychological balance and a sense of agency. Although the indirect effect (*a* × *b* = 0.42) accounted for most of the total effect, the direct path from bullying to retaliation remained significant (*β* = 0.23, *t* = 4.82), showing that both immediate emotional arousal and long-term internalized oppression contribute to the emergence of retaliatory responses. These results suggest that retaliation is not simply an impulsive reaction but rather a form of adaptive resistance to enduring social injustice and identity invalidation. The findings deepen the interpretation of minority stress, showing that it functions not only as a personal psychological burden but also as an outcome of social reproduction that reinforces inequality through emotional and cognitive mechanisms. For SGM autistic undergraduates, the accumulation of stigma and exclusion fosters a dual condition of isolation and emotional fatigue, making retaliation a symbolic effort to reclaim dignity and control in contexts of persistent disempowerment. This dynamic underscores the limitations of current university anti-bullying strategies that focus narrowly on behavior management while overlooking the chronic psychological strain borne by intersectional minorities. Effective intervention therefore requires a shift from reactive control to preventive transformation—reducing systemic bias, alleviating identity exposure anxiety, and enhancing peer inclusion and institutional support. Addressing these deeper psychosocial dimensions is essential for mitigating the indirect effects of minority stress on retaliatory impulses and for cultivating an academic environment grounded in structural justice and psychological safety (see Table 6, Figure 4).

**Table 6:** Moderation by SGM status and multi-group SEM comparison.

Variable	*β*	*t*	*p*	Sig
Bullying (X)	0.27	5.67	<0.001	***
Minority stress (M)	0.52	10.84	<0.001	***
SGM status (SGM)	0.19	4.11	<0.001	***
M × SGM interaction	0.11	2.24	*p* = 0.027	*

#### 4.5.2 Multi-group SEM comparison

The multigroup structural equation modeling results (Table 7) revealed significant differences between SGM and heterosexual autistic undergraduates in the links between bullying, minority stress, and retaliatory impulses [Δ*χ*^2^(3) = 14.19, *p* < 0.001], underscoring SGM status as a defining structural factor. Among heterosexual students, bullying significantly predicted minority stress (*β* = 0.60, *p* < 0.001) and retaliatory impulses (*β* = 0.18, *p* < 0.01), with minority stress mediating the relationship (*β* = 0.57, *p* < 0.001). For SGM students, all coefficients were stronger (0.72, 0.66, and 0.26, respectively; all *p* < 0.001), showing that bullying experiences more readily translate into chronic stress and reactive emotions. This intensified linkage reflects how overlapping stigmas associated with neurodivergent traits and sexual minority identities undermine emotional regulation and resilience., making external prejudice more directly internalized as tension and hostility. These patterns should not be viewed as personal weakness but as evidence of the psychological imprint of structural inequality. When institutional and peer support are limited, accumulated distress often manifests as defensiveness or is misinterpreted as behavioral dysfunction.

**Table 7:** Multi-group structural equation model (SEM) comparison results.

Group	X → M	M → Y	X → Y	Δ*χ*^2^(3)	*p*	Sig
Heterosexual	0.60***	0.57***	0.18**	—	—	—
SGM	0.72***	0.66***	0.26***	14.19	<0.001	***

*p < 0.10, **p < 0.05, ***p < 0.01.

Compared with heterosexual peers, who benefit from greater social validation and emotional resources, SGM students face a thinner protective network, leaving the stress–emotion–behavior chain more fragile and reactive. This reveals that retaliation among marginalized students often functions as a defensive expression of injustice rather than a symptom of aggression. The findings suggest that current interventions focusing on individual emotion regulation are insufficient. Instead, universities should strengthen inclusion policies, peer education, and culturally sensitive counseling to reduce the structural reproduction of stress and retaliation. Retaliatory impulses thus mirror the broader dynamics of exclusion, implying that breaking the bullying–stress–retaliation cycle requires not only psychological support but also systemic change toward equity and acceptance.

### 4.6 Robustness analyses

#### 4.6.1 Robustness with control variables

**Figure 5:**
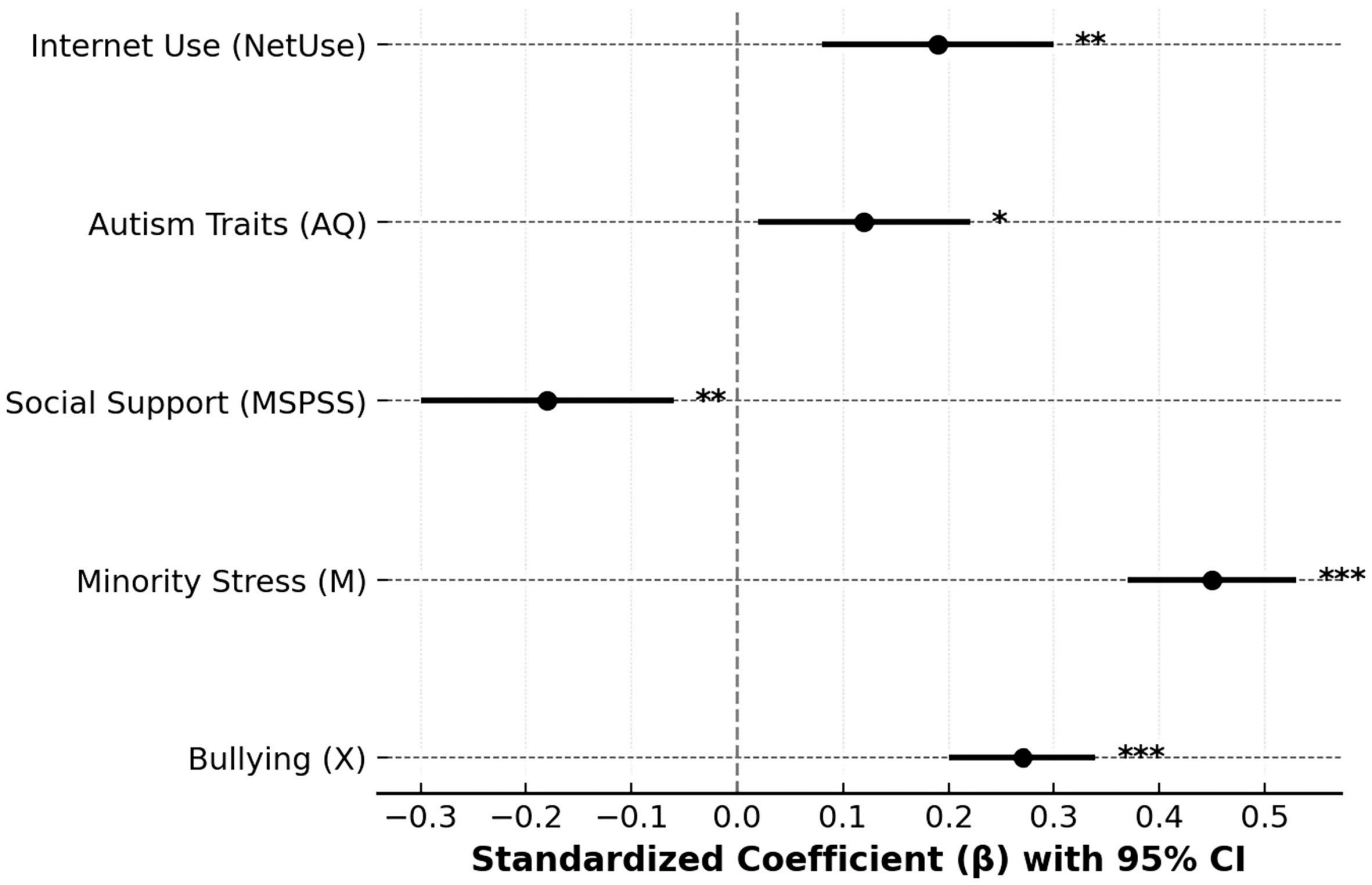
Coefficient forest plot for robustness test with control variables.

Table 8, Figure 5 present the results of the robustness analysis after controlling for gender, academic year, autistic traits, social support, and online engagement, showing that the core relationships of the model remained significant and consistent in direction. Bullying (*β* = 0.27, *p* < 0.001) and minority stress (*β* = 0.45, *p* < 0.001) continued to significantly predict retaliatory impulses, indicating that the link between social exclusion and emotional reactivity is structurally stable and not easily altered by individual or contextual factors. This suggests that retaliatory impulses are not merely situational emotional outbursts but reflect a psychological pattern shaped by enduring social inequality. High levels of minority stress imply that experiences of bullying and discrimination become internalized as feelings of insecurity and social devaluation, reinforcing hostile attribution and defensive cognition. Conversely, the negative coefficient for social support (*β* = −0.18, *p* = 0.003) highlights its protective role in buffering stress and reducing antagonistic responses. When individuals lack supportive resources, negative emotions are more easily internalized and transformed into defensive impulses. The positive effects of autistic traits (*β* = 0.12, *p* = 0.022) and online engagement (*β* = 0.19, *p* = 0.007) further suggest amplification effects, as individuals with higher autistic characteristics may misinterpret ambiguous cues as threats, and frequent online exposure can intensify negative affect through social comparison and emotional contagion, forming a continuous “online reinforcement–offline reaction” loop.

**Table 8:** Robustness test with control variables.

Control variable	*β*	*p*	Sig
Bullying (X)	0.27	<0.001	***
Minority stress (M)	0.45	<0.001	***
Social support (MSPSS)	−0.18	0.003	**
Autism traits (AQ)	0.12	0.022	*
Internet use (NetUse)	0.19	0.007	**

*p < 0.10, **p < 0.05, ***p < 0.01.

At a deeper level, the regression forest plot in Figure 5 shows that minority stress had the highest standardized coefficient and the narrowest confidence interval, confirming its central role in shaping retaliatory impulses. Even when overt bullying decreases, the persistence of internalized stigma and identity anxiety can sustain emotional tension and defensive responses. This finding underscores the structural nature of psychological strain: when bias and exclusion remain embedded in the environment, individuals’ affective systems adapt toward vigilance and resistance. Retaliatory impulses, therefore, are not expressions of personal fragility but psychological reflections of systemic inequality. Heterosexual autistic students showed lower retaliatory impulses under similar stress conditions, likely due to higher levels of acceptance and social connectedness, whereas SGM autistic students—facing dual exclusion—had fewer recovery resources and more intense emotional responses. The findings call for a shift in mental health interventions within universities: rather than framing emotional reactivity as an individual dysfunction, institutions should address its social origins. Building inclusive and affirming environments, reducing stigma, and enhancing access to supportive networks are essential strategies to disrupt the stress–impulse pathway. In this sense, psychological education should evolve from individual correction toward structural reform, creating a sustainable and equitable support system that recognizes the realities of intersectional minority students.

#### 4.6.2 Bootstrap validation

Table 9 presents the indirect effect estimates based on 500 bootstrap resamples. The mediating pathway from bullying to retaliatory impulses through minority stress remained significant and stable [*β* = 0.42, 95% CI (0.37, 0.49), *p* < 0.001], indicating strong statistical robustness. The confidence interval did not cross zero across resamples, underscoring the central role of minority stress in the bullying–retaliation linkage. These results suggest that the identified mechanism does not depend on particular sample features or measurement artifacts. Rather, it reflects a general psychosocial pattern among autistic undergraduates: bullying reliably activates minority stress, which in turn heightens emotional reactivity and retaliatory tendencies. The stability of this indirect effect strengthens the structural connection between social exclusion and psychological conflict and clarifies how social inequality is reproduced through psychological pathways. In conjunction with the multi-group and moderation findings reported earlier, the bootstrap evidence confirms that sexual minority autistic students exhibit more sensitive and rapidly accumulating stress responses along this chain. This difference is unlikely to be accidental and is consistent with the long-term psychological consequences of social marginalization at the group level. From a practical standpoint, the findings highlight the persistence and systemic nature of the psychological consequences of campus bullying and social exclusion, which are unlikely to be resolved through short-term individual interventions alone. Effective protection requires institutional and cultural changes that foster an inclusive campus climate, reduce the spread of identity stigma, and strengthen networks that promote psychological safety in order to disrupt the bullying–stress–retaliation pathway. The high consistency of the bootstrap results also provides a firm statistical basis for future intervention models, indicating that the mechanism is reproducible and generalizable under varying sampling and measurement conditions. In sum, the mediated pathway withstands statistical scrutiny and, in social terms, shows how marginalized identities perpetuate inequality through implicit psychological processes. These findings offer actionable empirical support for university mental health policies and anti-discrimination initiatives.

**Table 9:** Bootstrap validation results.

Bootstrap samples	Effect type	*β* (indirect)	95% CI (lower–upper)	*p*	Sig	Interpretation
500	Indirect effect	0.42	[0.37, 0.49]	<0.001	***	Indirect path remains significant and stable under Bootstrap resampling, confirming robustness.

*p < 0.10, **p < 0.05, ***p < 0.01.

### 4.7 Exploratory analyses

#### 4.7.1 Effects of gender, grade, and autism traits

**Figure 6:**
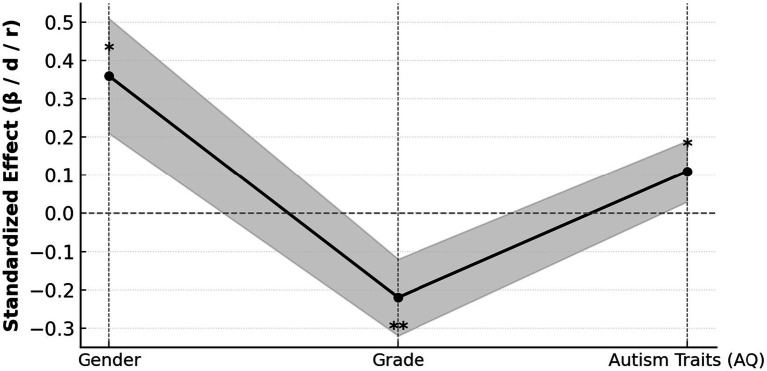
Standardized effects of gender, grade, and autism traits on revenge intention (with 95% CI).

Table 10, Figure 6 reveal that gender, academic year, and autistic traits each exerted a significant influence on retaliatory psychological responses, reflecting how socialized expectations and neurodevelopmental characteristics interact in shaping emotional behavior. Male autistic undergraduates reported higher scores for retaliatory aggression than females (*d* = 0.36, *p* = 0.021), indicating that men tend to externalize emotional reactions such as anger and confrontation when experiencing bullying or injustice, whereas women are more inclined to internalize distress. This divergence is not merely a matter of biological difference but mirrors the social construction of gender norms: men are often permitted or even encouraged to express anger, while women are socialized to display restraint and emotional control. Within autistic populations, where emotional sensitivity and difficulties in interpreting social cues are already heightened, these gendered expectations become further amplified. The interaction between gender norms and neurodivergent traits intensifies polarization in emotional expression, producing two distinct defensive strategies—outward resistance among men and inward withdrawal among women. This suggests that retaliatory responses in autistic individuals cannot be viewed in isolation from broader cultural scripts that regulate emotion and social behavior.

**Table 10:** Effects of gender, grade, and autism traits.

Variable	Path	Coefficient (*β*/*d*)	*p*	Sig
Gender	Males scored higher on revenge aggression (*d* = 0.36)	0.36	0.021	*
Grade	Higher grade → lower bullying experience (*r* = −0.22)	−0.22	0.002	**
Autism traits (AQ)	→ Revenge intention (*β* = 0.11)	0.11	0.024	*

*p < 0.10, **p < 0.05, ***p < 0.01.

Academic year differences reveal another dimension of developmental adaptation. Senior students reported significantly fewer bullying experiences (*r* = −0.22, *p* = 0.002), suggesting that with increased experience and identity consolidation, autistic students gradually develop more effective social navigation strategies, reducing their exposure to exclusion and conflict. This pattern highlights the existence of an “adaptation threshold” in university socialization: early undergraduate years may represent a critical period when the lack of established peer networks and uncertainty in identity make students especially vulnerable to social rejection. The positive predictive effect of autistic traits on retaliatory intention (*β* = 0.11, *p* = 0.024) further underscores the amplifying role of neurodivergent traits in stress responses, as difficulties in emotion recognition and intention inference may heighten threat perception and defensive tendencies. The standardized effects plot shows that gender had the strongest influence, academic year exerted a negative effect, and autistic traits had a positive effect, forming a multidimensional interplay among gender, development, and cognition. These findings underscore that retaliatory emotions among autistic students are not mere behavioral irregularities but reflect cumulative social exclusion and learned defense patterns. Therefore, effective intervention must go beyond behavior regulation to address structural inequities. Universities should develop inclusive environments that acknowledge gendered and neurodiverse emotional needs, provide targeted counseling for first-year adaptation, and foster peer education that normalizes difference. By integrating social inclusion with psychological support, institutions can help autistic students achieve greater emotional balance, reduce retaliatory tension, and cultivate a campus culture rooted in empathy and equity.

#### 4.7.2 Potential roles of social support and internet use intensity

Table 11 identifies potential mechanisms involving social support and intensity of internet use. Social support significantly and negatively predicted minority stress (*β* = −0.28, *p* < 0.001), whereas intensity of internet use significantly and positively predicted retaliatory impulses (*β* = 0.21, *p* = 0.001). These findings indicate protective and risk functions within the psychological response system. Higher levels of social support effectively alleviate the burden associated with bullying and identity-based discrimination, helping autistic undergraduates maintain psychological stability when facing external prejudice. Support comes not only from family or close relationships but also from peers and faculty who convey acceptance and understanding. It buffers emotional processing and reduces feelings of isolation and identity threat. By contrast, greater intensity of internet use correlates with stronger retaliatory impulses, suggesting a “double-edged” role of digital environments in emotion regulation for this group. Online platforms can provide alternative spaces for social connection and identity expression, yet anonymity and emotional contagion may amplify anger, anxiety, and hostility, making impulsive reactions more likely under provocation. The combined pattern in the figures suggests a complementary risk profile: when real-world support is scarce, individuals may turn to the internet for emotional sustenance, but fragmented and highly exposed online interactions can heighten stress and trigger affective cycles. These results imply that the mental health of autistic students cannot rely solely on individual emotion-regulation strategies; it also depends on systemic social support. Creating inclusive, safe, and stable interpersonal contexts, expanding opportunities for face-to-face interaction, and strengthening campus governance of online harassment can reduce the external conditions that foster retaliatory tendencies. In practice, universities should recognize that access to social support functions as a psychological defense resource rather than merely emotional comfort. Without guidance, high-intensity internet use may become a medium for reproducing psychological stress. Enhancing offline support, promoting healthy online engagement, and building positive community environments are therefore key strategies for weakening the stress chain and preventing impulsive responses.

**Table 11:** Potential roles of social support and internet use intensity.

Variable	Path	*β*	*p*	Sig
Social support (MSPSS)	→ Minority stress	−0.28	<0.001	***
Internet use (NetUse)	→ Revenge impulsivity	0.21	0.001	**

## 5 Discussion

### 5.1 Psychological genesis of minority stress triggered by bullying: identity threat and the cumulative effects of social exclusion

Given the cross-sectional design, the associations reported here are interpreted as an explanatory pattern consistent with MST and social information processing accounts, rather than as evidence of definitive causal effects. This study found that bullying experiences were significantly and positively associated with minority stress [*β* = 0.69, *t* = 12.48, 95% CI (0.58, 0.77), *p* < 0.001], with the model explaining 56% of the variance (*R*^2^ = 0.56). The result points to the structural and persistent impact of social exclusion on the mental health of autistic undergraduates. It is consistent with a core proposition of MST (11): external prejudice, social exclusion, and institutional neglect are linked to longer-term psychological burden through internalized stigma and identity-related anxiety. In this context, bullying is not only an interpersonal harm but also a mechanism of social reproduction. It becomes routinized through seemingly benign practices such as “jokes,” exclusion, and silence, and is gradually tolerated by institutional and cultural norms. When individuals remain in marginalized positions, identity threat may be activated (44). Their interpretations of social interactions may become more negative, giving rise to defensive cognitive patterns marked by expectations of rejection and self-blame. In our data, bullying frequency was strongly correlated with self-reported internalized stigma (*r* = 0.58, *p* < 0.001), indicating a feedback loop between external discrimination and internal self-reproach. Within China’s collectivist culture, the social logics of harmony and face can obscure the legitimacy of difference, leading autistic students to attribute bullying to being “not good enough” or “not fitting in,” which may intensify shame and social withdrawal. Public discourse often combines surface-level inclusion with latent exclusion, making the pressure more covert.

When educational institutions treat assimilation as the benchmark of successful adaptation, they implicitly reshape the selves of those who differ, demanding “integration” while eroding the right to express difference. In such a structural environment, bullying is not merely a problem of individual behavior; it reflects the system’s limited understanding of diversity and underlying power asymmetries. Williams et al. (45) argue that prolonged social exclusion is associated with chronic psychological stress and heightened sensitivity to hostility. Our results are consistent with this account: under conditions of limited social support, autistic undergraduates may be more prone to a sustained state of heightened vigilance and defense. The relationship between bullying and minority stress is thus not a one-way causal line but a process through which social structure may continually reproduce individual vulnerability. Practically, understanding this mechanism may prompt universities and policymakers to reassess existing inclusion frameworks. Effective anti-bullying efforts may benefit from moving beyond “zero-tolerance” rhetoric to address educational structures and cultural norms, building safeguards informed by neurodiversity perspectives and identity-related equity considerations. Only when the focus shifts from “asking them to adapt” to “changing our own exclusionary mechanisms” will the pressure chain forged by identity threat and social exclusion begin to break.

### 5.2 The mediating role of minority stress: cognitive pathways of hostile attribution and defensive processing

Given the cross-sectional design, the associations reported here are interpreted as an explanatory pattern consistent with Minority Stress Theory and Social Information Processing accounts, rather than as evidence of definitive causal effects. This study further supports a partial mediating role of minority stress in the association between bullying and retaliatory impulses [indirect effect *β* = 0.42, 95% CI (0.36, 0.50), *p* < 0.001]. Bullying was positively associated with minority stress (*β* = 0.69, *p* < 0.001), and minority stress was in turn associated with retaliatory impulses (*β* = 0.61, *p* < 0.001), underscoring psychological stress as a central explanatory link between social exclusion and affective–behavioral responses. This pattern is consistent with MST (11) regarding the internalization of discrimination and aligns with Social Information Processing theory (28) across groups. As an external social stimulus, bullying may heighten threat perception. Through internalized stigma and identity-related anxiety, minority stress appears to channel this heightened sensitivity into hostile processing tendencies, forming a defensive cognitive pathway. Along this pathway, individuals tend to display systematic biases in attention to and interpretation of social cues; ambiguous cues are more readily construed as hostile, which may foster retaliatory affect and impulsive reactions. The Dual-Mode SIP Model proposed by Verhoef et al. (30) posits that individuals confronted with social threat may respond via a fast, affective route or regulate responses through a reflective, cognitive route. In our sample, higher scores on retaliatory impulses were associated with greater reliance on the affective route, suggesting that prolonged social exclusion may weaken inhibitory control and allow emotion-driven processing to dominate decision-making.

The mediating role of minority stress reflects the interaction between social structure and cognitive processing. Discrimination and exclusion do not directly produce aggression; rather, they are associated with changes in interpretive models of the social world, such that hostile responses become integrated into psychological defense. In practice, this mechanism helps explain how inequality may be internalized and expressed through behavioral counteraction. For autistic and SGM undergraduates, persistent exclusion is linked to the construction of a world model organized around expectations of hostility, a form of defensive processing that may regulate affect in the short term but is also associated with intensified social isolation and cycles of conflict over time. From this perspective, retaliatory impulses should not be dismissed as simple failures of self-control. They can be understood as psychological echoes of structural violence. When social exclusion is normalized under the banners of competition or free speech, hostile reactions among stigmatized groups may represent emotional resistance to perceived injustice. Educational settings that overlook this mechanism and evaluate retaliation solely through disciplinary or moral lenses risk reinforcing inequality and deepening defensive cognition among minorities. Accordingly, campus interventions and anti-bullying policies may benefit from targeting cognitive processing: rebuilding safer interpretive frameworks, reducing hostile attribution bias, and pairing emotion-regulation training with peer-understanding initiatives and anti-discrimination education. Such measures may weaken the implicit chain from social stress to cognitive bias to retaliatory response and promote repair at both psychological and structural levels. At the same time, acknowledging the structural origins of retaliatory impulses does not imply that such responses are without social or psychological cost; persistent retaliatory tendencies may exacerbate interpersonal conflict, reinforce exclusion, and ultimately undermine individual well-being. At the same time, sustained retaliatory tendencies may also exacerbate interpersonal conflict, reinforce stigmatizing perceptions, and undermine long-term psychological well-being, underscoring the need for interventions that address both structural injustice and its behavioral consequences.

### 5.3 Dual moderation in the bullying–stress–retaliation chain: buffering by social support and amplification by internet use

Given the cross-sectional nature of the data, the associations observed here are interpreted as explanatory rather than causal. The results nevertheless show a clear pattern in which social support and internet use are differentially related to retaliatory affect. Higher perceived social support was associated with lower levels of retaliatory impulse (*β* = −0.18, *p* = 0.003), whereas greater intensity of internet use was associated with stronger negative emotional reactions (*β* = 0.21, *p* = 0.001). Together, these variables accounted for 56% of the variance in retaliatory impulse (*R*^2^ = 0.56), indicating that both interpersonal context and media engagement are closely linked to how stress is experienced and expressed. The association between social support and lower retaliatory affect is consistent with the idea that stable and affirming relationships can mitigate the psychological strain associated with discrimination, exclusion, and misunderstanding. Autistic undergraduates who perceive acceptance and understanding from family members, peers, or instructors appear less inclined to interpret prejudice as a reflection of personal inadequacy and more able to regulate their responses to adverse social experiences. In line with the buffering hypothesis proposed by (46), higher levels of social support were also associated with lower minority stress (*β* = −0.28, *p* < 0.001). These findings suggest that support functions not merely as emotional comfort but as a resource that helps contain emotional escalation following repeated experiences of marginalization. By contrast, the positive association between internet use intensity and retaliatory impulse points to potential risks associated with digitally mediated interaction. Although online spaces may offer opportunities for connection and self-expression, high levels of engagement in the absence of adequate offline support may coincide with increased exposure to hostile cues, emotional contagion, and social comparison. Participants reporting higher levels of internet use also reported stronger retaliatory impulses [*M* = 3.74 vs. 2.91, *t*(342) = 4.62, *p* < 0.001], suggesting that online contexts may amplify emotional reactivity under conditions of stress. Features such as anonymity and algorithm-driven content exposure may further complicate emotion regulation by intensifying perceived threat or injustice. When offline understanding and support are limited, online environments may therefore reinforce rather than alleviate distress. These findings indicate that responses to bullying and minority stress are shaped by a combination of individual, relational, and contextual factors. Efforts to reduce retaliatory impulses among autistic and SGM students may benefit from strengthening everyday sources of support within universities, while also attending to how digital environments interact with existing vulnerabilities. Rather than focusing exclusively on individual self-control or online behavior, interventions that address both interpersonal support and the broader social conditions in which online engagement occurs may be better aligned with the patterns observed here.

## 6 Contributions and limitation

Research on bullying, minority stress, and mental health among autistic university students has often addressed these processes separately, leaving limited empirical insight into how they are psychologically connected. The present study responds to this gap by examining how experiences of bullying are associated with emotional and cognitive responses through minority stress, drawing on both MST and Social Information Processing perspectives. Rather than framing stress or retaliatory impulse as isolated outcomes, the findings suggest that repeated social exclusion is linked to internalized stress processes that shape how individuals interpret and respond to interpersonal threat. This pattern appears particularly salient among neurodivergent students who also hold sexual or gender minority identities, highlighting the importance of intersectional approaches when examining vulnerability in higher education contexts.

The findings of this study offer several contributions while also requiring cautious interpretation in light of important methodological limitations. Given the cross-sectional design, all modeled associations should be understood as theoretically informed explanatory relationships rather than evidence of temporal ordering or causal directionality among bullying experiences, minority stress, and retaliatory impulses. All core variables were assessed using self-report measures collected within a single survey context, which raises the possibility of common method variance, including social desirability and shared measurement context effects. No formal *post hoc* statistical test (e.g., Harman’s single-factor test or marker-variable techniques) was conducted to quantify this risk. Nevertheless, several features of the results reduce the likelihood that the observed associations are solely attributable to a single common method factor. Specifically, the measurement model demonstrated clear discriminant validity across constructs, effect sizes varied substantially across structural paths, and theoretically meaningful moderation and group differences were observed—patterns that are difficult to explain by method bias alone. In addition, procedural precautions such as the use of validated multi-item scales, assured anonymity, and mixed item ordering were employed to mitigate method-related inflation. Despite these considerations, common method variance cannot be fully ruled out and should be taken into account when interpreting the magnitude of the reported associations. The sampling strategy also warrants caution. Participants were recruited via an online platform and eligibility criteria required recent experiences of bullying, which may have resulted in an over-representation of individuals experiencing elevated psychological distress. As such, the findings may not be fully generalizable to the broader population of autistic university students, particularly those with lower exposure to victimization or greater institutional support.

At a conceptual level, the study lends support to extending minority stress frameworks beyond sexual minority populations and underscores their relevance for understanding the experiences of neurodivergent students within institutional environments. By integrating minority stress processes with social information processing mechanisms, the findings illustrate how social contexts may be associated with changes in threat perception, attributional bias, and emotional regulation. From a practical standpoint, the results suggest that interventions focused solely on strengthening individual coping capacities may have limited effectiveness if broader social and institutional conditions remain unchanged. Although social support was associated with lower levels of stress, its effects varied across offline and digital contexts, indicating that peer norms, institutional practices, and online environments jointly shape students’ psychological experiences. Future research employing longitudinal designs, multiple measurement sources, and more diverse samples would be valuable for clarifying developmental dynamics, strengthening causal inference, and extending the generalizability of the present findings.

## Data Availability

The raw data supporting the conclusions of this article will be made available by the authors, without undue reservation.
